# Effects of Kangaroo Mother Care in the NICU on the Physiological Stress Parameters of Premature Infants: A Meta-Analysis of RCTs

**DOI:** 10.3390/ijerph19010583

**Published:** 2022-01-05

**Authors:** Delia Cristóbal Cañadas, Antonio Bonillo Perales, Rafael Galera Martínez, María del Pilar Casado-Belmonte, Tesifón Parrón Carreño

**Affiliations:** 1 Neonatal and Paediatric Intensive Care Unit, Torrecárdenas University Hospital, 04009 Almería, Spain; deliacristobal@gmail.com; 2 Pediatrics Department, Torrecárdenas University Hospital, 04005 Almería, Spain; abonillop@gmail.com (A.B.P.); galeramartinez@gmail.com (R.G.M.); 3 Department of Economics and Business, University of Almería, 04120 Almería, Spain; 4 Department of Nursing, Physiotherapy and Medicine, University of Almería, 04120 Almería, Spain; tpc468@ual.es; 5 Andalusian Council of Health at Almería Province, 04005 Almería, Spain

**Keywords:** kangaroo mother care, meta-analysis, neonatal intensive care, premature infant, physiological stress

## Abstract

Objective: The aim of this study was to analyze the randomised controlled trials that explored the effect of kangaroo mother care on physiological stress parameters of premature infants. Methods: Two independent researchers performed a systematic review of indexed studies in PubMed, Embase, CINAHL, Cochrane and Scopus. We included data from randomized controlled trials measuring the effects of kangaroo care compared to standard incubator care on physiological stress outcomes, defined as oxygen saturation, body temperature, heart rate and respiratory rate. The PRISMA model was used to conduct data extraction. We performed a narrative synthesis of all studies and a meta-analysis when data were available from multiple studies that compared the same physiological parameters with the kangaroo method as an intervention and controls and used the same outcome measures. Results: Twelve studies were eligible for inclusion in this meta-analysis. According to statistical analysis, the mean respiratory rate of preterm infants receiving KMC was lower than that of infants receiving standard incubator care (MD, −3.50; 95% CI, −5.17 to −1.83; *p* < 0.00001). Infants who received kangaroo mother care had a higher mean heart rate, oxygen saturation and temperature, although these results were not statistically significant. Conclusions: Current evidence suggests that kangaroo care in the neonatal intensive care unit setting is a safe method that may have a significant effect on some of the physiological parameters of stress in preterm infants. However, due to clinical heterogeneity, further studies are needed to assess the effects of physiological stress in the neonatal intensive care unit on the development of preterm infants.

## 1. Background

In the neonatal intensive care unit (NICU), preterm infants are exposed to stress factors such as invasive hospital procedures, bright light and noise from medical equipment [1,2]. Useful and indispensable interventions in the NICU can lead to physiological and behavioural reactions in preterm infants [3,4]. In addition, the separation of babies from their mothers and the neonatal intensive care unit environment itself limits visual, tactile and acoustic interactions between infants and mothers, affecting maternal bonding [5].

The World Health Organization has defined kangaroo mother care (KMC) as early, continuous and prolonged skin-to-skin contact between the mother and the newborn, with the father or parents involved as kangaroo care providers, as well as frequent and exclusive breastfeeding and early discharge from the hospital [6]. However, in neonatal intensive care, intermittent kangaroo mother care is typically used for short periods of time once or more per day for a variable number of days [7]. Kangaroo care provides babies with tactile stimulation through early contact of the mother’s skin with the infant, kinesthetic visual stimuli from direct skin contact, olfactory stimuli from suckling and motor stimuli from nipple sucking. It also promotes the interaction, bonding and attachment between infant and mother, which are essential for emotional and social development [8,9]. However, the optimal duration of kangaroo care has not yet been determined.

The expected benefits of kangaroo care include a stable heart rate, improved oxygen saturation and respiratory rate, improved lactation of the infant and increased milk production in the mother [5,10,11,12,13] and it is also often considered as a stress reducer intervention for infants in the neonatal intensive care unit [14] and positive effects on neurological, cognitive, emotional, behavioural and social development in the short and long term [15]. The kangaroo method is also considered an effective strategy to reduce procedural pain in premature infants [16].

Previous meta-analyses found that kangaroo mother care promotes early initiation of breastfeeding [17] and protects against many adverse neonatal outcomes [18]. In randomized controlled trials (RCTs) in healthy newborns, kangaroo care is also associated with improved breastfeeding and benefits outcomes in early mother-infant attachment, infant crying and cardiorespiratory stability [3]. Conde-Agudelo et al. [19] reviewed 16 Cochrane randomised clinical trials of kangaroo mother care, a strategy of continuous or intermittent skin-to-skin contact, full or near full breastfeeding and early discharge for infants weighing less than 2500 g at birth in resource-poor settings. They found that kangaroo mother care was associated with reduced mortality, reduced infections and sepsis at discharge, as well as reduced severe infections, hypothermia and length of hospital stay.

Considering the importance of the kangaroo method in neonatal intensive care and the evolution of premature infants and several more accurate clinical trials conducted on this topic in recent years, we decided to investigate the effectiveness of the kangaroo method in the neonatal intensive care unit setting of premature infants through a meta-analysis, with a risk of bias analysis and an extensive search method. The aim of this study was to conduct a meta-analysis of randomised controlled trials on the effects of kangaroo mother care on physiological stress parameters in preterm infants in neonatal intensive care compared to conventional neonatal care.

## 2. Patients and Methods

### 2.1. Search Strategy and Selection Criteria

The study followed the PRISMA (Preferred Reporting Items for Systematic Reviews and Meta-Analyses) guidelines [20]. We conducted a comprehensive literature search using PubMed, Embase, CINAHL, Scopus, Cochrane and Web of Science databases, with an initial search in March 2020 and a follow-up search in September 2020, without any language restrictions. The search was performed using the following keywords in combination: KMC, kangaroo mother care, kangaroo care, skin-to-skin contact, preterm, premature infant, stress, physiological parameters and autonomic nervous system. Where applicable, the above terms are used in the combination of “and” and “or” as specified by the search engine.

This study focused on the physiological measures of stress most commonly used in the experimental setting (neonatal intensive care). The results for the following parameters were extracted from the included studies: heart rate, respiratory rate, oxygen saturation and body temperature. These four measurements were analysed separately as they could not be combined into a single stress measurement. Other stress measures, such as cortisol, were excluded because there was insufficient evidence for the validity of these measures and their infrequent use did not allow for a meaningful meta-analysis. Studies for which we were unable to extract data were also considered as additional exclusion criteria.

### 2.2. Inclusion and Exclusion Criteria

Inclusion criteria were reports on the original research study comparing kangaroo mother care with conventional or incubator care in preterm infants in the neonatal intensive care unit and studies using any definition of kangaroo mother care with at least the skin-to-skin component and that would report on one of the physiological stress parameters (heart rate, respiratory rate, oxygen saturation or temperature) in the outcomes in premature infants. We excluded studies with non-human subjects and studies with less than ten participants and a lack of a comparison group without KMC. During a full-text review, articles that did not report the original results of the original research were excluded, if the test did not measure physiological outcomes of neonatal stress in kangaroo mother care compared to conventional care in neonatal intensive care units. Articles reporting the effects of kangaroo care were also excluded because the intervention during a painful or stressful procedure. We limited our analysis to studies of preterm infants with a gestational age of 37 weeks or less.

### 2.3. Data Extraction

In this study, Preferred Reporting Items for Systematic Reviews and Meta-Analyses (PRISMA) [20] were employed in order to identify relevant articles and inform about the selection process. All abstracts of the articles were selected by two independent reviewers. Where it was not clear from the abstract whether it was eligible for inclusion, the full text was reviewed. Data from all articles that met the inclusion criteria were then screened by two reviewers. At each stage, the two reviewers compared the results and reached a consensus. For those papers where important information was missing, authors were contacted by email to obtain the data. Information was collected about the research project, the characteristics of the participants, descriptions and times of kangaroo mother care and comparison groups, observation times, results, bias and association measures. We extracted estimates of relative risk (RR) or mean difference effect (MD) with a 95% confidence interval (CI). We collected data on exposure to kangaroo mother care components and the duration of practiced skin-to-skin contact. The extracted data were imported to Review Manager 5.3 by one reviewer.

### 2.4. Risk of Bias Assessment

The risk of bias of the included studies was evaluated using the Cochrane collaboration risk of bias tool [21]. The domains of the risk of bias tool are: “random sequence generation”; “allocation concealment”; “blinding of participants and personnel”; “blinding of outcome assessment”; “incomplete outcome data”; “selective reporting”. The risk of bias of each study result was deduced by defining the three main domains in the risk of bias tool: “random sequence generation”; “allocation concealment”; “blinding of outcome assessment”. If these three domains were low risk in a trial, the outcome of interest in that trial was considered low risk. If one domain was unclear or high risk, the outcome of interest in that study was considered unclear or high risk, respectively. To illustrate the risk associated with the bias graphs, Review Manager 5.3 was used. The risks of bias were evaluated by two authors and differences of opinion were resolved through discussion.

### 2.5. Statistical Analysis

To assess the effects of kangaroo mother care compared to conventional treatment on each neonatal physiological parameter, a meta-analysis was performed using the random effects model with the standardised mean differences. Using the Mantel–Haenszel method, typical RR estimates and 95% confidence intervals (CI) were obtained. Heterogeneity was determined using the Cochrane Q Chi-square test and I2 values. The effect size and Z-statistics were used to evaluate the overall effect. For the interpretation of the results of the meta-analysis, an SMD value of 0.2, 0.5 or 0.8 was considered to indicate a small, moderate or large effect size of the intervention, respectively. The random-effects method was used because of the high level of I2, which is an important statistic for assessing heterogeneity and clinical heterogeneity. Heterogeneity between studies was defined as low if I2 < 50%, medium if I2 = 50–75% and high if I2 > 75% Publication bias was assessed using Egger’s test and expressed using the funnel plot. Using Review Manager 5.3, standardised mean differences (SMDs) and 95% confidence intervals were estimated.

Subgroup analysis and investigation of heterogeneity. A subgroup analysis was planned for the primary outcomes. First, gestational ages (very preterm, 28 to 32 weeks) were compared to moderate to late preterm infants (more than 32 to 37 weeks). Second, the durations of intervention (kangaroo mother care, less than 60 min versus more than or equal to 60 min) were compared.

Sensitivity analysis. For our last two main results, there was a high level of heterogeneity; much of the variability was due to two studies [22,23], so we performed a sensitivity analysis excluding these two studies to examine the effects on the results and the high level of heterogeneity in EPIDAT 3.1 software. Due to the varying methodological quality of the studies, caution is advised when interpreting their results.

## 3. Results

### 3.1. Study Selection

**Figure 1:**
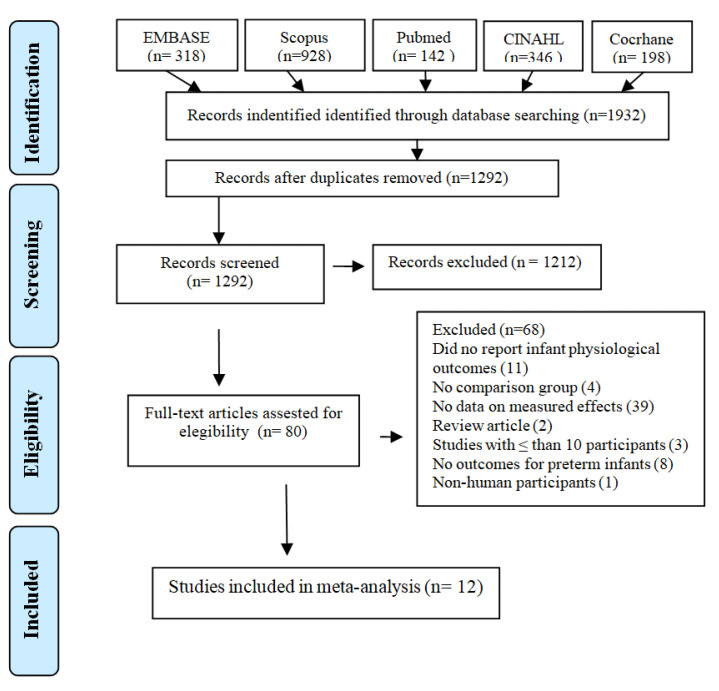
PRISMA flow diagram.

The search strategy yielded 1932 (out of which, 640 were marked as duplicates) studies. In total, 1292 records underwent abstract screening. Of those, 1212 did not meet inclusion criteria. Out of those, 80 full-text articles were assessed for eligibility, and among these 12 were included in the meta-analysis. The PRISMA flow diagram is presented in Figure 1.

### 3.2. Characteristics of Included Studies

The study characteristics are presented in Table 1. Twelve studies met the inclusion criteria. The total sample sizes in the studies ranged from 185 to 311 infants. Three studies were in the USA; two in Australia; two in South of Korea; and one each in Denmark, India, Brazil, Canada and Turkey. All articles were in English. The characteristics of the interventions differed significantly between the studies. Most of the studies were conducted with very preterm infants and four studies with late preterm infants. The mean gestational age of the included studies was 30.72 ± 2.64 and the mean standard deviation was 5.9. The mean weight of the infants was 1376.8 ± 295.49 g and the mean of the standard deviations was 315.74. The durations of kangaroo mother care ranged from approximately 30 min to a mean of 9.8 h.

### 3.3. Risk of Bias Assessment

**Figure 2:**
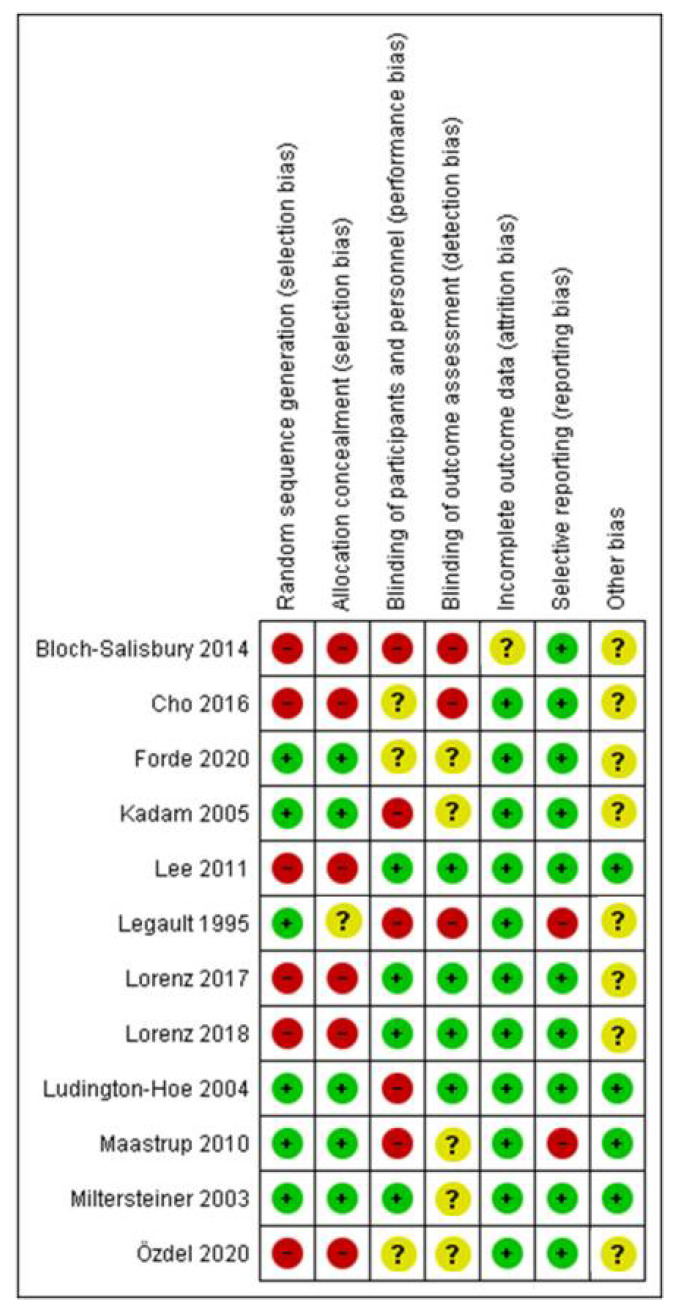
Summary of the risks of bias of the included studies.

**Figure 3:**
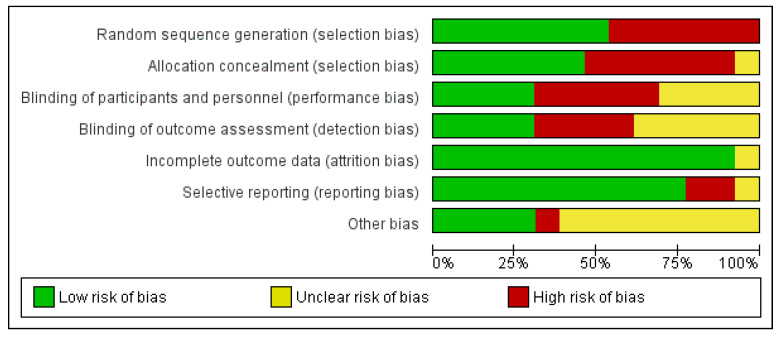
Risks of bias of the included studies.

The risks of bias of the included studies were assessed with the Cochrane collaboration risk of bias tool [21]. The risks of bias of each domain in all studies are summarized in Figure 2 and Figure 3. With regard to the overall risk of bias of an outcome in a study, 2 studies had low risk of bias [22,24], several studies had unclear risk of bias [25,26,27] and 7 studies had high risk of bias [9,23,28,29,30,31,32]. 

### 3.4. Meta-Analyses

All studies reviewed were randomised controlled trials. Where moderate (I2 between 30–50%) or large statistical heterogeneity (I2 greater than 50%) was observed, the text cautions against this, and caution should be exercised in the interpretation of these results showing an average treatment effect.

### 3.5. Heart Rate

**Figure 4:**
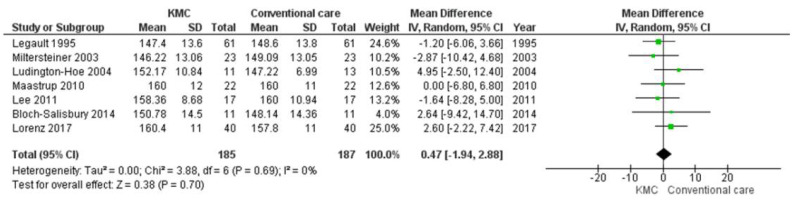
Forest plots for effects of KMC compared with conventional care on heart rate and respiratory rate (beats/min).

A total of eight independent studies were included in this analysis. Data from 372 infants were evaluated (185 in the intervention group and 187 in the control group). The mean heart rate of infants receiving conventional care was lower than that of infants receiving kangaroo mother care, although evidence of differences between groups did not reach statistical significance and there was no evidence of statistical heterogeneity in this outcome (MD 0.47 beats per minute (BPM), 95% CI −1.94 to 2.88); (heterogeneity: T² = 0.00, *p* = 0.69, I² 0%). For forest plots, see Figure 4.

### 3.6. Respiratory Rate

**Figure 5:**
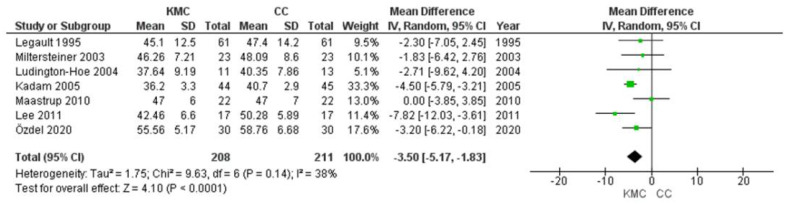
Forest plots for effects of KMC compared with conventional care on heart rate and respiratory rate (breaths/min).

Data from 419 infants were evaluated (208 in the intervention group and 211 in the control group). Figure 5 shows the results of a meta-analysis model including all seven studies. Overall, significant differences in respiratory rate values were observed in favour of the kangaroo mother’s care compared to the control group (MD, −3.50; 95% CI, −5.17 to −1.83; *p* < 0.00001). However, moderate heterogeneity was observed in these seven studies (I2 = 38%).

### 3.7. Oxygen Saturation

**Figure 6:**
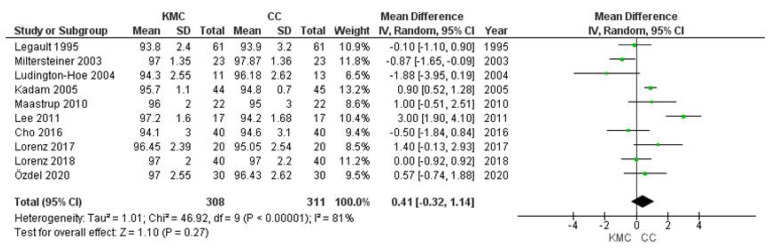
Forest plots for effects of KMC compared with conventional care on oxygen saturation (%).

Data on infant oxygen saturation were obtained from 10 studies. Data from 619 infants were evaluated (308 in the intervention group and 311 in the control group). The mean oxygen saturation was lower in infants receiving conventional care than in infants receiving kangaroo mother care, with statistically non-significant data on differences between groups and high heterogeneity (MD 0.41% 95% CI −0.32–1.14) in this result (heterogeneity: T² = 1.01, *p* < 0.00001, I² 81%) (Figure 6). This effect was similar in all subgroups of study characteristics, gestational age of infants and duration of intervention (Supplemental Figure S1).

The heterogeneity was mainly due to the findings of the Miltersteiner [22] and Lee [23] studies. We performed sensitivity analyses where results from these studies were excluded; for oxygen saturation the elimination of these two studies reduced the heterogeneity (heterogeneity Tau² = 0.32, *p* = 0.03, I² = 54%) (Supplemental Analysis S1), with an overall average sensitivity of −0.18 C.I. 95%, −0.14–0.50 (Supplemental Analysis S2).

### 3.8. Temperature

**Figure 7:**
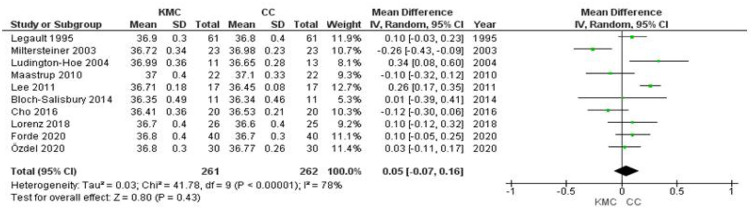
Forest plots for effects of KMC compared with conventional care on temperature (°C).

Data on infant oxygen saturation were obtained from 10 studies. Data from 523 infants were evaluated (261 in the intervention group and 262 in the control group). The mean body temperature of conventionally cared for infants was 0.05°C higher than in kangaroo mother care (95% CI −0.07 to 0.16; I² 78%) (Figure 7). This effect was similar in all subgroups of study characteristics, gestational age of infants and duration of intervention (Supplementary Figure S2). All preterm infants analysed had a body temperature range of 36.34–37.1 °C. The results of this meta-analysis should be interpreted with caution due to the heterogeneity and very small sample size.

The heterogeneity was mainly due to the findings of the Miltersteiner [22] and Lee [23] studies. We performed sensitivity analyses where results from these studies were excluded; for temperature the elimination of these two studies reduced the heterogeneity (heterogeneity Tau² = 0.01, *p* = 0.13, I² = 37%) (Supplemental Analysis S3), although the differences were not clinically significant, with an overall average sensitivity of −0.14 C.I. 95%, −0.08–0.38 (Supplemental Analysis S4).

## 4. Discussion

This meta-analysis examined the effects of the mother kangaroo method on physiological parameters of stress in preterm infants during neonatal intensive care unit hospitalization. Our main analysis of studies that evaluated physiological parameters of stress as an outcome in preterm infants in the NICU indicated a significant impact of the intervention on stress reduction after the kangaroo method intervention (*p* < 0.05) on respiratory rate, an effect that is likely to have clinical significance as an indicator of a stress response. Similar results were found by Pados and Hess [33], who investigated whether skin-to-skin care was an intervention used to reduce stress in the NICU and concluded that the research showed that skin-to-skin care resulted in short-term improvements in cardiorespiratory stress compared to incubator care. Although the effect on heart rate was not significant in the kangaroo intervention, there was no significant increase in heart rate, so taken together, these findings suggest that kangaroo therapy contributes to clinical stability. The mean infant axillary temperature difference of 0.05 °C was not found to be clinically significant. This is consistent with the findings of Conde-Agudelo [19], whereby preterm infants performing the kangaroo method were associated with a reduced incidence of hypothermia at discharge. Subgroup analyses in our study indicated that variations in temperature and oxygen saturation over the duration of the intervention and gestational age were not associated with an improvement in these parameters and there was no negative effect, even on very preterm infants. There is very little evidence to suggest that KMC has negative effects on stress in even the most fragile infants [33].

Although the results varied somewhat, the studies confirmed that kangaroo care of preterm infants reduces scores of physiological stress parameters compared to incubator or conventional care. No negative outcomes associated with skin-to-skin contact were reported in any of the included studies. Many studies showed no statistically significant differences in physiological variables, probably due to the sample size and control for other factors described above. We found that improvements in heart rate, respiratory rate, oxygen saturation and body temperature associated with maternal kangaroo care may be of modest clinical significance, although taken together they support the hypothesis that maternal kangaroo care is a safe method and may improve overall physiological regulation in infants, which may have important implications for other long-term outcomes. There is very little evidence that kangaroo care has a negative effect on neonatal stress, and kangaroo care should be considered an important part of optimal care of preterm infants in the neonatal unit.

In earlier similar studies, Boundy et al. [18] performed a systematic review and meta-analysis of randomised controlled trials and observational studies to assess the impacts of kangaroo mother care and on neonatal outcomes, regardless of birth weight or gestational age. Among low birth weight infants <2000 g, kangaroo mother care had no significant effect on mean heart rate, infants receiving kangaroo mother care had a respiratory rate 3 breaths per minute slower and oxygen saturation 0.9% higher than in controls, while a lower risk of hypothermia was associated with kangaroo mother care. Moore et al. [8] conducted a systematic review and meta-analysis to evaluate the impact of direct or early skin-to-skin contact versus traditional contact on initiation and maintenance of breastfeeding in healthy infants and infant physiology.

The mean heart rate was lower in control infants with the mother separated than in infants with skin-to-skin contact, although evidence of differences between the groups was not clinically significant and the results differed significantly between studies. For the respiratory rate, the results were in favour of infants with skin-to-skin contact and the results were clinically significant.

Our estimated effects were similar to those in the studies by Boundy et al. [18] and Moore et al. [8]. Differences between the results of this meta-analysis are explained by the inclusion of only randomised controlled trials, the gestational ages of the children and the inclusion of more recent studies in our study.

### Implications for Future Research

This review has identified the need for greater transparency in research reporting and several areas where more research is needed. More rigorously designed studies are needed, with larger samples and standardised outcome measures and careful consideration of the study design and confounding factors (e.g., stress-inducing procedures such as painful procedures). Transparent and comprehensive reporting of interventions and trial results is essential, as transparency allows for the replication and transfer of studies to clinical practice. Parallel-group randomised controlled trials could be conducted to assess the long-term effects of kangaroo care for preterm infants by extending the duration of the intervention after neonatal intensive care unit discharge, although this is currently a major gap in our knowledge. The separation of infant and parent due to intensive or prolonged hospitalisation may impede the development of healthy parent–infant relationships. Research is needed to assess the long-term impact of kangaroo care on the formation of healthy parent–infant bonds and secure attachment.

## 5. Conclusions

There is evidence that kangaroo mother care is similar to conventional care. Accordingly, it is a safe method that has positive effects on certain parameters of physiological stress compared to incubator care. The present meta-analysis suggests that KMC is not harmful to preterm infants as young as 28 weeks, can be provided for prolonged periods without compromising the infant and may have a positive impact on certain physiological markers in preterm infants in neonatal intensive care. Future research should include a more rigorous methodology, including more studies with sufficient power, randomisation of studies and adequate control groups.

## Figures and Tables

**Table 1:** Characteristics of the included studies.

Author, Year	Country	KMC and Comparison Group	N	Gestational Age Mean (SD) or Median (IQR)	Birth Weight (g)	KMC Duration	Physiologic Parameter Measured
Legault, 1995	Canada	KMC compared traditional method	61	30 (24–35)	1225 (685–1835)	30 min	HR, BR O2sat, Tº
Miltersteiner, 2003	Brazil	KMC compared incubator	23	34.1 ± 1.88	1740 ± 280.04	60 min	HR, BR O2sat, Tº
Ludington-Hoe, 2004	USA	KMC compared to standard care	24	(KMC) 33.55 ± 1.57	1467 ± 228	180 min	HR, BR O2sat, Tº
				(CC) 34.42 ± 1.08	14,611 ± 217		
Kadam, 2005	India	KMC compared to conventional method of care	89	(KMC) 33.3 ± 2.1	1467 ± 228	9.8 ± 3.7 h	BR, O2sat
				(CC) 34 ± 1.7	14,611± 217		
Maastrup, 2010	Denmark	Skin-to-skin-contact	22	25 (23–27)	735 (460–1050)	98 (51–387) min	HR, RR O2sat, Tº
Lee, 2011	South Korea	KMC compared to Conventional Care	34	(KMC) 27.50 ± 2.89	990 ± 360	30 min	HR, RR O2sat, Tº
				(CC) 29.87 ± 3.25	1180 ± 450		
Bloch-Salisbury, 2014	USA	Compared with control group in incubator	11	29.87 ± 2.74	1393 ± 127	41.3 ± 21.2 min	HR, RR O2sat, Tº
Cho, 2016	South Korea	KC compare with routine care	40	(KMC) 30.12 ± 16.29	1660.00 ± 225.20	30 min	HR, RR O2sat, Tº
				(CC) 28.81 ± 20.63	1442.00 ± 128.00		
Lorenz, 2017	Australia	SSC compared with incubator	40	27.6 (26.0–28.9)	969 (817–1263)	90 min	HR O2sat
Lorenz, 2018	Australia	SSC compared with incubator	40	30.6 (29.1–31.7)	1370 (1062–1572)	90 min	HR O2sat
Forde, 2020	USA	KMC versus incubator care	51	(KMC) 32.00 ± 2.6	1827 ± 492	60 min	Tº
				(CC) 31.40 ± 2.1	1642 ± 545		
Özdel, 2020	Turkey	Prone position and kangaroo care	30	30.20 ± 2.63	1455.43 ± 607.85 (593–3080)	180 min	HR, RR O2sat, Tº

KMC = Kangaroo mother care; SSC = Skin-to-skin contact; HR = Heart rate; RR = Respiratory rate; Tº = temperature; O2sat = oxygen saturation.

## Data Availability

The data that support the findings of this study are available from the corresponding author upon reasonable request.

## Supplementary Materials

The following are available online at https://www.mdpi.com/article/10.3390/ijerph19010583/s1. Figure S1: Subgroup analysis for the effects of kangaroo mother care compared with conventional care on oxygen saturation (%); Figure S2: Subgroup analysis for the effects of kangaroo mother care compared with conventional care on temperature (°C); Analysis S1: Oxygen saturation; Analysis S2: Oxygen saturation sensitivity analysis with influence plot; Analysis S3: Temperature; Analysis S4: Temperature sensitivity analysis with influence plot.

## References

[1] Gao H., Xu G., Gao H., Dong R., Fu H., Wang D., Zhang H., Zhang H. (2015). Effect of repeated Kangaroo Mother Care on repeated procedural pain in preterm infants: A randomized controlled trial. Int. J. Nurs. Stud..

[2] Cong X., Wu J., Vittner D., Xu W., Hussain N., Galvin S., Fitzsimons M., McGrath J., Henderson W. (2017). The impact of cumulative pain/stress on neurobehavioral development of preterm infants in the NICU. Early Hum. Dev..

[3] Blume-Peytavi U., Lavender T., Jenerowicz D., Ryumina I., Stalder J., Torrelo A., Cork M. (2016). Recommendations from a European Roundtable Meeting on Best Practice Healthy Infant Skin Care. Pediatric Dermatol..

[4] Medina I.M.F., Granero-Molina J., Fernández-Sola C., Hernández-Padilla J.M., Avila M.C., Rodríguez M.D.M.L. (2018). Bonding in neonatal intensive care units: Experiences of extremely preterm infants’ mothers. Women Birth.

[5] Cho E.-S., Kim S.-J., Kwon M.S., Cho H., Kim E.H., Jun E.-M., Lee S. (2016). The Effects of Kangaroo Care in the Neonatal Intensive Care Unit on the Physiological Functions of Preterm Infants, Maternal–Infant Attachment, and Maternal Stress. J. Pediatric Nurs..

[6] Chan G.J., Valsangkar B., Kajeepeta S., Boundy E.O., Wall S. (2016). What is kangaroo mother care? Systematic review of the literature. J. Glob. Health.

[7] Nyqvist K.H., Anderson G.C., Bergman N., Cattaneo A., Charpak N., DaVanzo R., Ewald U., Ibe O., Ludington-Hoe S., Mendoza S. (2010). Towards universal Kangaroo Mother Care: Recommendations and report from the First European conference and Seventh International Workshop on Kangaroo Mother Care. Acta Paediatr..

[8] Moore E.R., Anderson G.C., Bergman N., Dowswell T. (2012). Early skin-to-skin contact for mothers and their healthy newborn infants. Cochrane Database Syst. Rev..

[9] Luong K.C., Nguyen T.L., Thi D.H.H., Carrara H.P., Bergman N.J. (2015). Newly born low birthweight infants stabilise better in skin-to-skin contact than when separated from their mothers: A randomised controlled trial. Acta Paediatr..

[10] Ionio C., Ciuffo G., Landoni M. (2021). Parent–Infant Skin-to-Skin Contact and Stress Regulation: A Systematic Review of the Literature. Int. J. Environ. Res. Public Health.

[11] De Almeida H., Venancio S.I., Sanches M.T.C., Onuki D. (2010). The impact of kangaroo care on exclusive breastfeeding in low birth weight newborns. J. Pediatrics.

[12] Bieleninik Ł., Ettenberger M., Epstein S., Elefant C., Arnon S. (2021). Potential Psychological and Biological Mechanisms Underlying the Effectiveness of Neonatal Music Therapy during Kangaroo Mother Care for Preterm Infants and Their Parents. Int. J. Environ. Res. Public Health.

[13] Bera A., Ghosh J., Singh A.K., Hazra A., Som T., Munian D. (2014). Effect of Kangaroo mother care on vital physiological parameters of the low birth weight newborn. Indian J. Community Med..

[14] Pados B.F. (2019). Physiology of Stress and Use of Skin-to-Skin Care as a Stress-Reducing Intervention in the NICU. Nurs. Women’s Health.

[15] Charpak N., Tessier R., Ruiz J.G., Hernandez J.T., Uriza F., Villegas J., Nadeau L., Mercier C., Maheu F., Marin J. (2017). Twenty-year Follow-up of Kangaroo Mother Care Versus Traditional Care. Pediatrics.

[16] Campbell-Yeo M., Johnston C.C., Benoit B., Disher T., Caddell K., Vincer M., Walker C.-D., Latimer M., Streiner D.L., Inglis D. (2019). Sustained efficacy of kangaroo care for repeated painful procedures over neonatal intensive care unit hospitalization: A single-blind randomized controlled trial. Pain.

[17] Mekonnen A.G., Yehualashet S.S., Bayleyegn A.D. (2019). The effects of kangaroo mother care on the time to breastfeeding initiation among preterm and LBW infants: A meta-analysis of published studies. Int. Breastfeed. J..

[18] Boundy E.O., Dastjerdi R., Spiegelman D., Fawzi W.W., Missmer S.A., Lieberman E., Kajeepeta S., Wall S., Chan G.J. (2016). Kangaroo Mother Care and Neonatal Outcomes: A Meta-analysis. Pediatrics.

[19] Conde-Agudelo A., Belizán J.M. (2003). Kangaroo mother care to reduce morbidity and mortality in low birthweight infants. Cochrane Database Syst. Rev..

[20] Moher D., Liberati A., Tetzlaff J., Altman D.G. (2009). Preferred reporting items for systematic reviews and meta-analyses: The PRISMA statement. BMJ.

[21] Higgins J.P.T., Altman D.G., Gøtzsche P.C., Jüni P., Moher D., Oxman A.D., Savović J., Schulz K.F., Weeks L., Sterne J.A.C. (2011). The Cochrane Collaboration’s tool for assessing risk of bias in randomised trials. BMJ.

[22] Miltersteiner A.R., Miltersteiner D.R., Rech V.V., Molle L.D. (2003). Respostas fisiológicas da Posição Mãe-Canguru em bebês pré-termos, de baixo peso e ventilando espontaneamente. Rev. Bras. Saúde Matern. Infant..

[23] Lee J., Bang K.-S. (2011). The Effects of Kangaroo Care on Maternal Self-esteem and Premature Infants’ Physiological Stability. Korean J. Women Health Nurs..

[24] Ludington-Hoe S.M., Anderson G.C., Swinth J.Y., Thompson C., Hadeed A.J. (2004). Randomized Controlled Trial of Kangaroo Care: Cardiorespiratory and Thermal Effects on Healthy Preterm Infants. Neonatal Netw..

[25] Forde D., Deming D.D., Tan J.B., Phillips R.M., Fry-Bowers E.K., Barger M.K., Bahjri K., Angeles D.M., Boskovic D.S. (2020). Oxidative Stress Biomarker Decreased in Preterm Neonates Treated With Kangaroo Mother Care. Biol. Res. Nurs..

[26] Kadam S., Binoy S., Kanbur W., Mondkar J.A., Fernandez A. (2005). Feasibility of kangaroo mother care in Mumbai. Indian J. Pediatrics.

[27] Maastrup R., Greisen G. (2010). Extremely preterm infants tolerate skin-to-skin contact during the first weeks of life. Acta Paediatr..

[28] Bloch-Salisbury E., Zuzarte I., Indic P., Bednarek F., Paydarfar D. (2014). Kangaroo care: Cardio-respiratory relationships between the infant and caregiver. Early Hum. Dev..

[29] Legault M., Goulet C. (1995). Comparison of Kangaroo Traditional Methods of Removing Preterm Infants From Incubators. J. Obstet. Gynecol. Neonatal Nurs..

[30] Lorenz L., Dawson J., Jones H., Jacobs S.E., Cheong J., Donath S., Davis P.G., Kamlin C.O.F. (2017). Skin-to-skin care in preterm infants receiving respiratory support does not lead to physiological instability. Arch. Dis. Child. Fetal Neonatal Ed..

[31] Lorenz L., Marulli A., Dawson J., Owen L.S., Manley B.J., Donath S.M., Davis P.G., Kamlin C.O.F. (2017). Cerebral oxygenation during skin-to-skin care in preterm infants not receiving respiratory support. Arch. Dis. Child. Fetal Neonatal Ed..

[32] Özdel D., Sarı H.Y. (2020). Effects of the prone position and kangaroo care on gastric residual volume, vital signs and comfort in preterm infants. Jpn. J. Nurs. Sci..

[33] Pados B., Hess F. (2020). Systematic Review of the Effects of Skin-to-Skin Care on Short-Term Physiologic Stress Outcomes in Preterm Infants in the Neonatal Intensive Care Unit. Adv. Neonatal Care.

