# Supplementary material for: Effects of Kangaroo Mother Care in the NICU on the Physiological Stress Parameters of Premature Infants: A Meta-Analysis of RCTs
Source: Int J Environ Res Public Health. 2022 Jan 5;19(1):583. doi: 10.3390/ijerph19010583 (PMC8744895; doi:10.3390/ijerph19010583)

Supplemental Figure S1. Subgroup Analysis and for the Effect of Kangaroo Mother Care Compared With Conventional Care on Oxygen Saturation, %

Very preterm Infants

Study or Subgroup	KMC Mean	KMC SD	KMC Total	CC Mean	CC SD	CC Total	Weight	Mean Difference IV, Random, 95% CI	Year
Legault 1995	93.8	2.4	61	93.9	3.2	61	15.7%	-0.10 [-1.10, 0.90]	1995
Maastrup 2010	96	2	22	95	3	22	12.8%	1.00 [-0.51, 2.51]	2010
Lee 2011	97.2	1.6	17	94.2	1.68	17	15.1%	3.00 [1.90, 4.10]	2011
Cho 2016	94.1	3	40	94.6	3.1	40	13.8%	-0.50 [-1.84, 0.84]	2016
Lorenz 2017	96.45	2.39	20	95.05	2.54	20	12.7%	1.40 [-0.13, 2.93]	2017
Lorenz 2018	97	2	40	97	2.2	40	16.1%	0.00 [-0.92, 0.92]	2018
Özdel 2020	97	2.55	30	96.43	2.62	30	13.9%	0.57 [-0.74, 1.88]	2020
**Total (95% CI)**			**230**			**230**	**100.0%**	**0.75 [-0.19, 1.69]**	

Heterogeneity: Tau² = 1.21; Chi² = 25.45, df = 6 (P = 0.0003); I² = 76%
Test for overall effect: Z = 1.57 (P = 0.12)

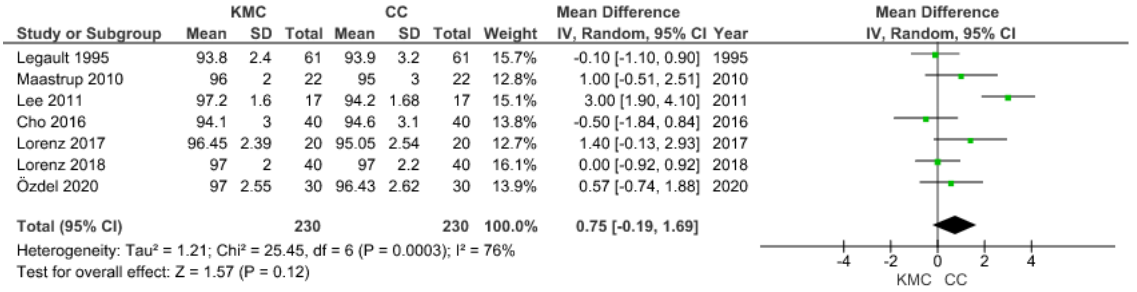

Preterm infants

Study or Subgroup	KMC Mean	KMC SD	KMC Total	CC Mean	CC SD	CC Total	Weight	Mean Difference IV, Random, 95% CI	Year
Miltersteiner 2003	97	1.35	23	97.87	1.36	23	36.7%	-0.87 [-1.65, -0.09]	2003
Ludington-Hoe 2004	94.3	2.55	11	96.18	2.62	13	23.9%	-1.88 [-3.95, 0.19]	2004
Kadam 2005	95.7	1.1	44	94.8	0.7	45	39.4%	0.90 [0.52, 1.28]	2005
**Total (95% CI)**			**78**			**81**	**100.0%**	**-0.42 [-2.01, 1.18]**	

Heterogeneity: Tau² = 1.64; Chi² = 20.98, df = 2 (P < 0.0001); I² = 90%
Test for overall effect: Z = 0.51 (P = 0.61)

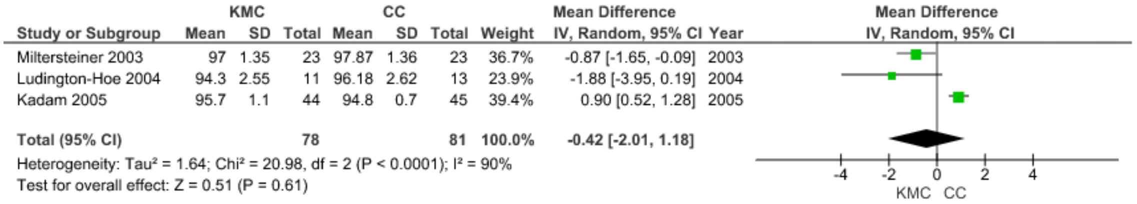

Duration of intervention

< 60 minutes.

Study or Subgroup	KMC Mean	KMC SD	KMC Total	CC Mean	CC SD	CC Total	Weight	Mean Difference IV, Random, 95% CI	Year
Legault 1995	93.8	2.4	61	93.9	3.2	61	34.1%	-0.10 [-1.10, 0.90]	1995
Lee 2011	97.2	1.6	17	94.2	1.68	17	33.6%	3.00 [1.90, 4.10]	2011
Cho 2016	94.1	3	40	94.6	3.1	40	32.3%	-0.50 [-1.84, 0.84]	2016
**Total (95% CI)**			**118**			**118**	**100.0%**	**0.81 [-1.37, 2.99]**	

Heterogeneity: Tau² = 3.37; Chi² = 21.95, df = 2 (P < 0.0001); I² = 91%
Test for overall effect: Z = 0.73 (P = 0.47)

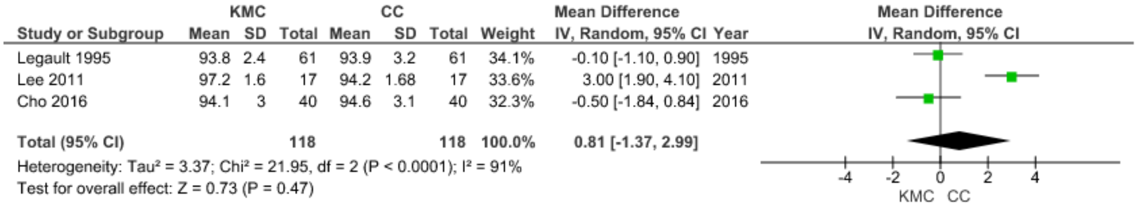

≥60 minutes

Study or Subgroup	KMC Mean	KMC SD	KMC Total	CC Mean	CC SD	CC Total	Weight	Mean Difference IV, Random, 95% CI	Year
Miltersteiner 2003	97	1.35	23	97.87	1.36	23	17.8%	-0.87 [-1.65, -0.09]	2003
Ludington-Hoe 2004	94.3	2.55	11	96.18	2.62	13	8.3%	-1.88 [-3.95, 0.19]	2004
Kadam 2005	95.7	1.1	44	94.8	0.7	45	20.7%	0.90 [0.52, 1.28]	2005
Maastrup 2010	96	2	22	95	3	22	11.8%	1.00 [-0.51, 2.51]	2010
Lorenz 2017	96.45	2.39	20	95.05	2.54	20	11.6%	1.40 [-0.13, 2.93]	2017
Lorenz 2018	97	2	40	97	2.2	40	16.6%	0.00 [-0.92, 0.92]	2018
Özdel 2020	97	2.55	30	96.43	2.62	30	13.3%	0.57 [-0.74, 1.88]	2020
**Total (95% CI)**			**190**			**193**	**100.0%**	**0.23 [-0.53, 0.99]**	

Heterogeneity: Tau² = 0.69; Chi² = 23.88, df = 6 (P = 0.0005); I² = 75%
Test for overall effect: Z = 0.60 (P = 0.55)

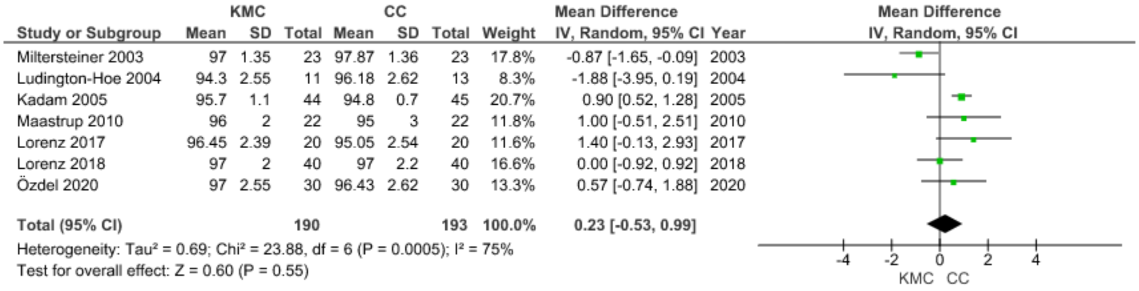

Supplemental Analysis S1. Oxygen Saturation

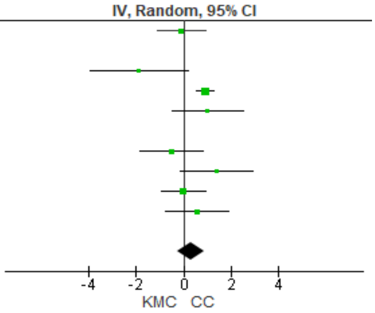

Study or Subgroup	KMC Mean	KMC SD	KMC Total	CC Mean	CC SD	CC Total	Weight	Mean Difference IV, Random, 95% CI	Year
Legault 1995	93.8	2.4	61	93.9	3.2	61	14.5%	-0.10 [-1.10, 0.90]	1995
Miltersteiner 2003	97	1.35	23	97.87	1.36	23	0.0%	-0.87 [-1.65, -0.09]	2003
Ludington-Hoe 2004	94.3	2.55	11	96.18	2.62	13	5.9%	-1.88 [-3.95, 0.19]	2004
Kadam 2005	95.7	1.1	44	94.8	0.7	45	23.7%	0.90 [0.52, 1.28]	2005
Maastrup 2010	96	2	22	95	3	22	9.3%	1.00 [-0.51, 2.51]	2010
Lee 2011	97.2	1.6	17	94.2	1.68	17	0.0%	3.00 [1.90, 4.10]	2011
Cho 2016	94.1	3	40	94.6	3.1	40	10.8%	-0.50 [-1.84, 0.84]	2016
Lorenz 2017	96.45	2.39	20	95.05	2.54	20	9.1%	1.40 [-0.13, 2.93]	2017
Lorenz 2018	97	2	40	97	2.2	40	15.7%	0.00 [-0.92, 0.92]	2018
Özdel 2020	97	2.55	30	96.43	2.62	30	11.1%	0.57 [-0.74, 1.88]	2020
**Total (95% CI)**			**268**			**271**	**100.0%**	**0.32 [-0.25, 0.89]**	

Heterogeneity: Tau² = 0.32; Chi² = 15.26, df = 7 (P = 0.03); I² = 54%
Test for overall effect: Z = 1.10 (P = 0.27)

Supplemental Analysis S2 Oxygen Saturation Sensitivity analysis with Influence Plot

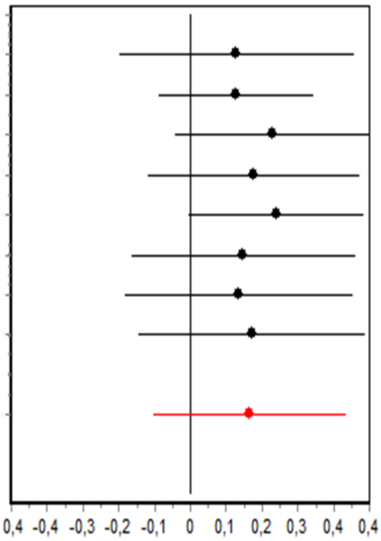

Study	(Year)	(n)	Sensitivity (95% confidence interval)
Legault	1995	417	0,215 (-0,166 -- 0,597)
Ludington-Hoe	2004	515	0,261 (-0,055 -- 0,578)
Kadam	2005	450	0,050 (-0,182 -- 0,283)
Maastrup	2010	495	0,151 (-0,211 -- 0,514)
Cho	2016	459	0.234 (-0,125 -- 0,595)
Lorenz	2017	499	0,132 (-0,220 -- 0,485)
Lorenz	2018	459	0,206 (-0,170 -- 0,584)
Ozdel	2020	479	0,172 (-0,202 -- 0,547)
GLOBAL		539	0,180 (-0,144 -- 0,506)

Supplemental Analysis S3.Temperature

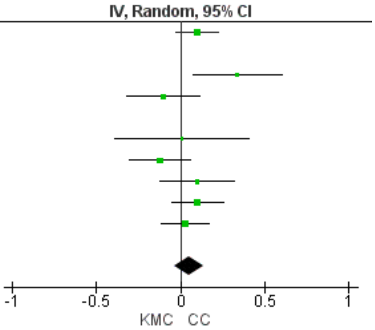

Study or Subgroup	KMC Mean	KMC SD	KMC Total	CC Mean	CC SD	CC Total	Weight	Mean Difference IV, Random, 95% CI	Year
Legault 1995	36.9	0.3	61	36.8	0.4	61	19.8%	0.10 [-0.03, 0.23]	1995
Miltersteiner 2003	36.72	0.34	23	36.98	0.23	23	0.0%	-0.26 [-0.43, -0.09]	2003
Ludington-Hoe 2004	36.99	0.36	11	36.65	0.28	13	8.1%	0.34 [0.08, 0.60]	2004
Maastrup 2010	37	0.4	22	37.1	0.33	22	10.6%	-0.10 [-0.32, 0.12]	2010
Lee 2011	36.71	0.18	17	36.45	0.08	17	0.0%	0.26 [0.17, 0.35]	2011
Bloch-Salisbury 2014	36.35	0.49	11	36.34	0.46	11	4.0%	0.01 [-0.39, 0.41]	2014
Cho 2016	36.41	0.36	20	36.53	0.21	20	13.3%	-0.12 [-0.30, 0.06]	2016
Lorenz 2018	36.7	0.4	26	36.6	0.4	25	10.4%	0.10 [-0.12, 0.32]	2018
Forde 2020	36.8	0.4	40	36.7	0.3	40	16.1%	0.10 [-0.05, 0.25]	2020
Özdel 2020	36.8	0.3	30	36.77	0.26	30	17.6%	0.03 [-0.11, 0.17]	2020
**Total (95% CI)**			**221**			**222**	**100.0%**	**0.05 [-0.03, 0.14]**	

Heterogeneity: Tau² = 0.01; Chi² = 11.20, df = 7 (P = 0.13); I² = 37%
Test for overall effect: Z = 1.22 (P = 0.22)

Supplemental Analysis S4.Temperature Sensitivity analysis with Influence Plot

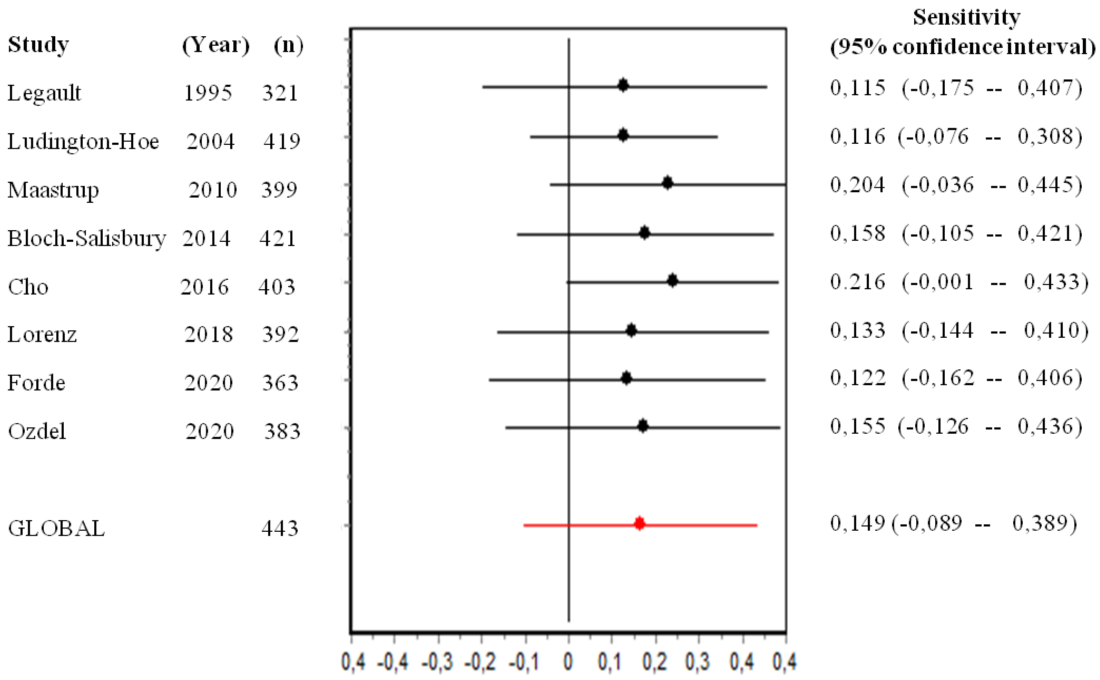

Study	(Year)	(n)	Sensitivity (95% confidence interval)
Legault	1995	321	0,115 (-0,175 -- 0,407)
Ludington-Hoe	2004	419	0,116 (-0,076 -- 0,308)
Maastrup	2010	399	0,204 (-0,036 -- 0,445)
Bloch-Salisbury	2014	421	0,158 (-0,105 -- 0,421)
Cho	2016	403	0.216 (-0,001 -- 0,433)
Lorenz	2018	392	0,133 (-0,144 -- 0,410)
Forde	2020	363	0,122 (-0,162 -- 0,406)
Ozdel	2020	383	0,155 (-0,126 -- 0,436)
GLOBAL		443	0,149 (-0,089 -- 0,389)

Supplemental Figure S2. Subgroup Analysis and for the Effect of Kangaroo Mother Care Compared With Conventional Care on Temperature, ℃.

Very preterm Infants

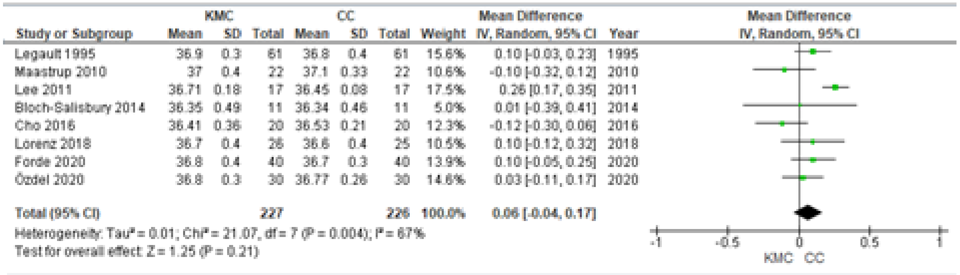

Study or Subgroup	KMC Mean	KMC SD	KMC Total	CC Mean	CC SD	CC Total	Weight	Mean Difference IV, Random, 95% CI	Year
Legault 1995	36.9	0.3	61	36.8	0.4	61	15.6%	0.10 [-0.03, 0.23]	1995
Maastrup 2010	37	0.4	22	37.1	0.33	22	10.6%	-0.10 [-0.32, 0.12]	2010
Lee 2011	36.71	0.18	17	36.45	0.08	17	17.5%	0.26 [0.17, 0.35]	2011
Bloch-Salisbury 2014	36.35	0.49	11	36.34	0.46	11	5.0%	0.01 [-0.39, 0.41]	2014
Cho 2016	36.41	0.36	20	36.53	0.21	20	12.3%	-0.12 [-0.30, 0.06]	2016
Lorenz 2018	36.7	0.4	26	36.6	0.4	25	10.5%	0.10 [-0.12, 0.32]	2018
Forde 2020	36.8	0.4	40	36.7	0.3	40	13.9%	0.10 [-0.05, 0.25]	2020
Özdel 2020	36.8	0.3	30	36.77	0.26	30	14.6%	0.03 [-0.11, 0.17]	2020
**Total (95% CI)**			**227**			**226**	**100.0%**	**0.06 [-0.04, 0.17]**	

Heterogeneity: Tau² = 0.01; Chi² = 21.07, df = 7 (P = 0.004); I² = 67%
Test for overall effect: Z = 1.25 (P = 0.21)

Preterm infants

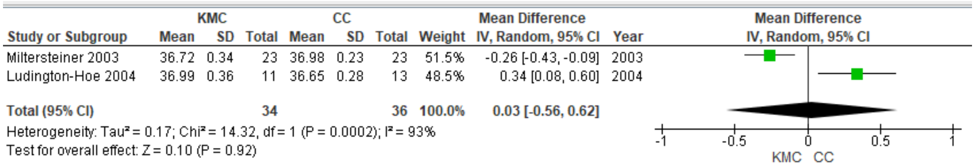

Study or Subgroup	KMC Mean	KMC SD	KMC Total	CC Mean	CC SD	CC Total	Weight	Mean Difference IV, Random, 95% CI	Year
Miltersteiner 2003	36.72	0.34	23	36.98	0.23	23	51.5%	-0.26 [-0.43, -0.09]	2003
Ludington-Hoe 2004	36.99	0.36	11	36.65	0.28	13	48.5%	0.34 [0.08, 0.60]	2004
Total (95% CI)			34			36	100.0%	0.03 [-0.56, 0.62]	

Heterogeneity: Tau² = 0.17; Chi² = 14.32, df = 1 (P = 0.0002); I² = 93%
Test for overall effect: Z = 0.10 (P = 0.92)

< 60 minutes.

Study or Subgroup	KMC Mean	KMC SD	KMC Total	CC Mean	CC SD	CC Total	Weight	Mean Difference IV, Random, 95% CI	Year
Legault 1995	36.9	0.3	61	36.8	0.4	61	29.8%	0.10 [-0.03, 0.23]	1995
Lee 2011	36.71	0.18	17	36.45	0.08	17	31.9%	0.26 [0.17, 0.35]	2011
Bloch-Salisbury 2014	36.35	0.49	11	36.34	0.46	11	12.8%	0.01 [-0.39, 0.41]	2014
Cho 2016	36.41	0.36	20	36.53	0.21	20	25.6%	-0.12 [-0.30, 0.06]	2016
**Total (95% CI)**			**109**			**109**	**100.0%**	**0.08 [-0.09, 0.26]**	

Heterogeneity: Tau² = 0.02; Chi² = 14.78, df = 3 (P = 0.002); I² = 80%
Test for overall effect: Z = 0.92 (P = 0.36)

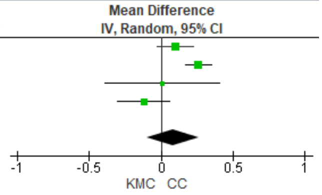

≥60 minutes

Study or Subgroup	KMC Mean	KMC SD	KMC Total	CC Mean	CC SD	CC Total	Weight	Mean Difference IV, Random, 95% CI	Year
Miltersteiner 2003	36.72	0.34	23	36.98	0.23	23	17.9%	-0.26 [-0.43, -0.09]	2003
Ludington-Hoe 2004	36.99	0.36	11	36.65	0.28	13	13.5%	0.34 [0.08, 0.60]	2004
Maastrup 2010	37	0.4	22	37.1	0.33	22	15.5%	-0.10 [-0.32, 0.12]	2010
Lorenz 2018	36.7	0.4	26	36.6	0.4	25	15.4%	0.10 [-0.12, 0.32]	2018
Forde 2020	36.8	0.4	40	36.7	0.3	40	18.5%	0.10 [-0.05, 0.25]	2020
Özdel 2020	36.8	0.3	30	36.77	0.26	30	19.1%	0.03 [-0.11, 0.17]	2020
**Total (95% CI)**			**152**			**153**	**100.0%**	**0.02 [-0.13, 0.17]**	

Heterogeneity: Tau² = 0.02; Chi² = 19.07, df = 5 (P = 0.002); I² = 74%
Test for overall effect: Z = 0.31 (P = 0.76)

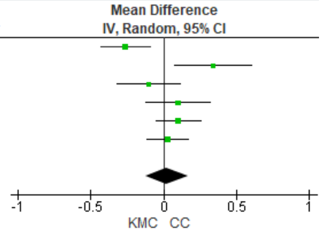

Supplement: Supplementary file 1 [file ijerph-19-00583-s001.zip › ijerph-1498166-supplementary.pdf]
